# Flavored Melons

**Published:** 1884-12

**Authors:** 


### FLAVORED MELONS.

OUR Pacific Coast contemporaries are heralding a dangerous line of experiments in flavoring melons: The *Los Angeles Herald*, a short time since, mentioned a new pr cess of flavoring melons by inserting a strip of cloth in the stem of the melon and immersing one end of the cloth in strawberry, vanilla, peppermint, sherry or some other popular flavors. The San Barnardino Index, a temperance paper, tells how the thing was tried in that town. He took lemon in his: "Mr. George Cooley, who lives on D street, some days ago having a fine, large melon that was ripening nicely, determined to try the flavoring idea. Lemon was selected as being most easily recognized. Yesterday the melon was cut and tested. The flavor was delicate, but easily recognized, and the new process unanimously pronounced a decided improvement." The next thing we know our Benegrass neighbors will be grafting one end of the rag to a still or raw liquor tank and be growing full-fledged mint juleps and cocktails in their watermelon patches.

**Electro-Medical Instruments.**—For many years, the electro-medical instruments manufactured by the Jerome Kidder M'f'g. Co. have enjoyed an enviable reputation as being the best adapted to the uses for which they are intended, of any made. Recently this company has exhibited at the Electrical Expositions, some new forms of apparatus, which have excited universal admiration for compactness, neatness, and beautiful workmanship. We have used these instruments in our own practice and can honestly say that for ease of adjusment and fine gradation of current they are without an equal. The tipping battery is a happy conception and removes the great source of inconvenience due to spilling of acid. Although we heartily recommend these instruments to users of electricity, the wide reputation they have acquired is sufficient guarantee of their excellence.

**The Sun as a Soporific.**—Sleepless people—and there are many of them in America—should court the sun. The very worst soporific is laudanum and the very best is sunshine. Therefore it is plain that poor sleepers should pass as many hours of the day in the sunshine and as few in the shade as possible. The injurious effects of shade is very noticeable in plants growing in secluded places, and ladies who are accustomed to carrying sunshades. The invigorating power of sunlight is infinite, and he whose skin is tawny seldom requires a pill.

**To Remove Foreign Bodies from the Eye**.—Before resorting to any metallic instrument for this purpose, Dr. C. D. Agnew (*American Practitioner*) would advise you to use an instrument made in the following manner: Take a splinter of soft wood, pine or cedar, and whittle it into the shape of a probe, making it about the length of an ordinary dressing probe. Then take a small, loose flock of cotton, and, laying it upon your forefinger, place the pointed end of the stick in the centre of it. Then turn the flock of cotton over the end of the stick, winding it round and round so as to make it adhere firmly. If you will look at the end of such a probe with a two-inch lens you will see that it is quite rough, the fibres of cotton making a file-like extremity, in the midst of which a little interstices. As the material is soft, it will do no harm to the cornea when brushed over its surface. When ready to remove the foreign body, have the patient rest his head against your chest, draw the upper lid up with the forefinger of your left hand, and press the lower lid down with the middle finger, and then delicately sweep the surface in which the foreign body is embedded with the end of the cotton probe. When the foreign body is lodged in the centre of the cornea, it is most important not to break up the external elastic lamina; for if you do, opacity may follow, and the slightest opacity in the centre of the cornea will cause a serious diminution in the sharpness of vision.

The *Lancet* reports a lecture on tea and coffee, in which people are advised to put the coffee for breakfast in an earthenware vessel, pour cold water over it, let it stand over night, and bring it to the boiling point by placing it in a water bath or double boiler in the morning, thus preserving the aroma. As the editor pronounces the lecture as being "perhaps the most brilliant since the series was begun," the writer has no doubt tried the plan.

This is the law of benefits between men: the one ought to forget at once what he has given, and the other ought never to forget what he has received.

Strength of character is not mere strength of feeling. It is the resolute restraint of strong feeling. It is unyielding resistance to whatever would disconcert us from without or unsettle us from within.

Discretion and hardy valor are the twins of honor, and nursed together make a conqueror; divided, a mere talker.

**Valerian for Superficial Wounds.**—At a recent meeting of the Societe de Biologie, M. Arragon brought forward a new method of dressing wounds, by which he declared their healing was hastened and the pain was made to disappear at once. The method consisted in the application of compresses wet with a decoction of thirty parts of valerian root in one thousand parts of water. Of fifty patients treated in this way, with only two had benefit failed to result, whether the wounds were lacerated or contused, but it is expressly stated that the treatment is of no avail in deep wounds. In one instance warm injections of the decoction were used for otitis media. The anodyne effect is attributed to the action of the valerianic acid on the terminal nerves, and an antiseptic influence also is credited to the remedy.

**Preservation of Dead Bodies.**—Edward I., who died in 1307, was found not decayed 463 years subsequently. The flesh on the face was a little wasted, but not putrid. The body of Canute, who died in 1017, was found fresh in 1766. That of William the Conqueror and his wife were perfect in 1522. In 1569 three Roman soldiers, in the dress of their country, fully equipped with arms, were dug out of a peat-moss near Aberdeen. They were quite fresh and plump after a lapse of about fifteen hundred years. In 1717 the bodies of Lady Kilsyth and her infant were emboweled and embalmed. Every feature and every limb was full. The infant's features were as composed as if he had only been asleep for eighty years. His color was as fresh and his flesh as plump aud fuli as in the perfect glow of health; the smile of infancy and innocency was on his lips. At a little distance it was difficult to distinguish whether Lady Kilsyth was alive or dead.

"Death is beautiful," says Chateaubriand, "it is our friend; nevertheless we are not grateful to it, because it presents itself to us masked, and its mask frightens us."

Self-distrust is the cause of most of our failures. In the assurance of strength there is strength, and they are the weakest, however strong, who have no faith in themselves or their powers.

At first I felt in uttermost despair,
And said, "O Lord, this cross I cannot bear."
But I have borne it, and I bear it now,
Only, oh only, do not ask me how.—*The Spectator.*

**Practical Science.**—European naturalists regard the attention paid in this country to economic entomology, and the aid that has been given it by various States and the general Government, as one sign of "a practical people." With all the specialization in instruction in the foreign universities, we are not aware that there is more than one which supports a professorship of entomology. This is Oxford, where the venerable Prof. Westwood honors the Hope foundation. In this country Harvard and Cornell each have their full professorship of this science, and to the latter a summer school, having special reference to agricultural entomology, has now been attached. This seems more appropriate than any of the summer schools now so much in vogue, inasmuch as the objects of study are at this season in the height of their investigations into the power of crops to sustain insect life. To further the interests of the school, the trustees of Cornell University have relieved Prof. Comstock of his duties during the winter; and an unusually good opportunity is thus afforded to teachers, as well as others, to familiarize themselves with the principles of this branch of economic science.

**Pernicious Castile Soap.**—The "Edinburgh Review" has started a crusade against castile soap, saying that there is very little doubt that even the very best brands of white castile soap are made from rancid olive oil, which, being of too poor quality for table use, is used for making soap. The best imported castile soap costs the importer only from ten to twelve cents a pound, all over that paid by the public being profit to the importer and retailer; and pure sweet olive oil brings too much to enable it to be made into castile soap and sold at any such price.

YOUNG WIFE—"My dear, you were the stroke oar at college, weren't you?"

"Yes, love."

"And a very prominent member of the gymnasium class?"

"I was the leader."

"And quite a hand at all athletic contests?"

"Quite a hand? My gracious! I was the champion walker, the best runner, the head man at lifting heavy weights, and as for carrying! why I could shoulder a barrel of flour and—"

"Well, love, just please carry the baby a couple of hours. I'm tired of it."

**The office of Hall's Journal of Health has been removed to Nos. 75 & 77 Barclay Street, New York, where all communications must hereafter be addressed.**

